# 

**Published:** 1875-08

**Authors:** 


					﻿Vol. 22.
AUGUST, 1875.
No 8.
TWENTY-SECOND YEAR
HALL’S
Journal of Health.
“The serial, a portion of which appears in this number, entitled “OUR
Summer at Maplewood,” has, been duly copyrighted according to law.”
PUBLISHED BY THE AMERICAN AND FOREIGN
PUBLICATION CO., 137 EIGHTH ST., NEW YORK.
▲cents for the Trade :
AMERICAN NEWS COMPANY, 121 NASSAU STREET.
NEW YORK NEWS COMPANY, 18 BEEKMAN STREET.
Term*, Two Dollars a Tear.
SINGLE NUMBER, 20 CENTS.
				

